# Online prevention programmes for university students: stakeholder perspectives from six European countries

**DOI:** 10.1093/eurpub/ckab040

**Published:** 2021-07-07

**Authors:** Madeleine Irish, Stefanie Kuso, Monika Simek, Michael Zeiler, Rachel Potterton, Peter Musiat, Martina Nitsch, Gudrun Wagner, Andreas Karwautz, Felix Bolinski, Eirini Karyotaki, Carla Soler Rovira, Ernestina Etchemendy, Rocio Herrero, Adriana Mira, Giulia Cormo, Rosa Baños, Azucena Garcia-Palacios, David D Ebert, Marvin Franke, Anna-Carlotta Zarski, Kiona Weisel, Thomas Berger, Michelle Dey, Michael P Schaub, Corinna Jacobi, Cristina Botella, Elia Oliver, Gemma Gordon, Lucy Spencer, Karin Waldherr, Ulrike Schmidt

**Affiliations:** 1 King's College London, Institute of Psychiatry, Psychology and Neuroscience, London, UK; 2 Ferdinand Porsche FernFH—Distance Learning University of Applied Sciences, Wiener Neustadt, Austria; 3 Department of Child and Adolescent Psychiatry, Medical University of Vienna, Vienna, Austria; 4 Department of Clinical, Neuro and Developmental Psychology, Vrije Universiteit Amsterdam, Amsterdam, The Netherlands; 5 Jaume I Universitat, Castellon, Spain; 6 CIBER Pathophysiology of Obesity and Nutrition (CB06/03), Carlos III Institute of Health, Madrid, Spain; 7 Department of Personality, Evaluation and Psychological Treatments, University of Valencia, Valencia, Spain; 8 Department of Clinical Psychology and Psychotherapy, Friedrich-Alexander-Universität Erlangen-Nürnberg, Erlangen, Germany; 9 Department of Clinical Psychology and Psychotherapy, Universität Bern, Bern, Switzerland; 10 Universität Zürich, Schweizer Institut für Sucht- und Gesundheitsforschung, Zurich, Switzerland; 11 Department of Psychology, Technische Universität Dresden, School of Science, Chair of Clinical Psychology and E-Mental-Health, Dresden, Germany; 12 South London and Maudsley NHS Foundation Trust, London, UK

## Abstract

**Background:**

Students beginning university are at a heightened risk for developing mental health disorders. Online prevention and early intervention programmes targeting mental health have the potential to reduce this risk, however, previous research has shown uptake to be rather poor. Understanding university stakeholders’ (e.g. governing level and delivery staff [DS] and students) views and attitudes towards such online prevention programmes could help with their development, implementation and dissemination within university settings.

**Methods:**

Semi-structured interviews, focus groups and online surveys were completed with staff at a governing level, university students and DS (i.e. student health or teaching staff) from six European countries. They were asked about their experiences with, and needs and attitudes towards, online prevention programmes, as well as the factors that influence the translation of these programmes into real-world settings. Results were analyzed using thematic analysis.

**Results:**

Participating stakeholders knew little about online prevention programmes for university settings; however, they viewed them as acceptable. The main themes to emerge were the basic conditions and content of the programmes, the awareness and engagement, the resources needed, the usability and the responsibility and ongoing efforts to increase reach.

**Conclusions:**

Overall, although these stakeholders had little knowledge about online prevention programmes, they were open to the idea of introducing them. They could see the potential benefits that these programmes might bring to a university setting as a whole and the individual students and staff members.

## Introduction

Emerging adulthood is associated with an increased incidence of mental health problems.^1^ This critical developmental stage coincides with the period where many people make the transition from school to higher education, which brings about additional stresses and lifestyle changes^2^ (e.g. increased independence, social network changes and academic challenges). In a recent representative survey, 27% of UK university students reported having a mental health problem, with depression, anxiety and eating disorders most commonly endorsed.^3^ Amongst students symptom-free prior to university, 9% became depressed and 20% became anxious at a clinically significant level by the middle of second year.^4^

Untreated mental health problems can have a significant impact on students’ social and academic outcomes.^5^^,^^6^ Help-seeking behaviours among this population, however, still remain relatively low.^7^^,^^8^ A systematic review of qualitative research found stigma and lack of trust to be the most frequently mentioned barriers to help-seeking. Additionally, long wait-lists seen in university counselling services and the difficulty in continuing therapy if splitting your time between two residences (e.g. family and university homes) are contributing factors.^7^^,^^9^ One potential way to overcome these barriers is through the use of online interventions that can potentially reduce stigma, address barriers to access and improve reach.

Online prevention programmes aim to prevent the potential development of mental health problems or the progression of subclinical symptoms by delivering interactive and tailored information and exercises related to mental health via computer, tablet or smartphone. Such programmes can either be universal (targeting whole populations) or indicated (targeting people who are at an increased risk of developing a mental health disorder).^10^ They usually can also involve interaction with coaches, moderators or experts as well as with other participants via chat functions, emails or discussion forums. Online prevention programmes for mental health problems show effectiveness both in general population samples and in higher education students.^11^^,^^12^ Universities are ‘settings for health’ according to the World Health Organization (WHO) as students spend a lot of time in the university and can be easily reached^13^ via the university setting. Therefore, they are seen as a good implementation setting for health promotion, prevention or early intervention.^14^^,^^15^ Furthermore, Internet-based interventions are considered as appropriate for students because the Internet is highly accessible and they also use it for seeking health-related information.^12^ Despite these advantages, uptake and retention rates for online prevention programmes are relatively poor,^12^^,^^16^^,^^17^ and it is unclear whether dropout predicts outcomes in online programmes.^18^

To increase the uptake of online programmes amongst students and improve implementation and maintenance in the university setting, it is essential to understand the benefits and barriers to their use. An online survey found that people were less likely to engage with online interventions compared with face-to-face treatment, in the main because there is no personal contact and they are perceived as less helpful.^16^ Topooco et al.^19^ found similar responses from a range of stakeholders, however neither study was specific to a student population and preventive interventions. When asking a student population, positive attitudes were shown towards an online mental health clinic, as it was seen as a convenient ‘first point of call’.^20^ Less clear are the university personnel and stakeholder views on prevention programmes in particular.

The aim of the current study was to explore stakeholders’ knowledge, attitudes and needs regarding online prevention programmes in the field of mental health for university students, as well as possible hindering and fostering contextual parameters for their widespread and sustained implementation and dissemination across six European countries. The goal was to understand stakeholder views to inform intervention adaptation, evaluation and delivery in the future.

## Methods

The current study was part of the ICARE stakeholder survey (https://www.icare-online.eu). A full description of the overall study design and methods has been published in a separate paper within this special issue.^21^

### Study design

Researchers approached three stakeholder groups:^21^ Target group (university students), potential facilitators/delivery staff (DS) (e.g. lecturers, university psychological services and student representatives) and governing level/policymakers (e.g. deans of education, senior university health care representatives). Due to the range of stakeholders and their specific characteristics, a mixed-methods approach was used and different survey methods were applied for each stakeholder group. For university students at least 18 years of age, focus groups were conducted. For stakeholders at a governing level, semi-structured interviews were conducted, because we assumed that persons in higher positions could be best approached via interview. Representatives of DS completed anonymous online questionnaires in order to reach a larger number of stakeholders. Online questionnaires and topic guides were strongly oriented towards the RE-AIM framework,^22^ which helps to develop programmes and disseminate and implement them in real-world settings by focusing on how to improve reach (R) and representativeness, produce meaningful outcomes (E—efficacy/effectiveness), improve adoption (A) by universities, enhance implementation of programmes (I) and maintain improvements in mental health, and sustain programmes over a longer term (M). Therefore, the pre-defined areas of questions for all stakeholders were determined by the following research questions: which (i) *experiences*, (ii*) needs*, (iii) *values and attitudes* do the different stakeholder groups have regarding online interventions to prevent mental health problems from being implemented into the existing healthcare systems and into specific settings like schools and universities? (iv) Which population groups are considered to be *underserved* regarding such interventions? and (v) Which *hindering and fostering context factors* need to be considered to optimize *reach, adoption, implementation* and *maintenance* when implementing online interventions to prevent mental health problems into the existing healthcare systems and into school and university settings? A description of the development of instruments can be found in the overall study design paper,^21^ and a copy of the topic guides (interviews and focus groups) and questionnaires can be found in Supplementary material.

Ethical approval for this study was obtained from the relevant local ethics committees for all participating consortium partners. Informed consent (including consent for audio recordings of focus groups and interviews) was obtained from all stakeholders.

### Recruitment and procedure

Stakeholders from the United Kingdom, Germany, Switzerland, Austria, Spain and the Netherlands were approached. The numbers of planned interviews, questionnaires and focus groups was determined *a priori* considering the concept of data saturation and experts’ recommendations and standards for the field.^21^ We planned a minimum of two focus groups per country. Undergraduate university students, mainly first year students, were recruited for the focus groups via university-wide email circulars, social media, online forums, word of mouth and flyers. Students studying either at the universities where the researchers are located or at other universities in the same city were invited to participate in the focus groups. The respective university towns were Amsterdam (Vrije Universiteit Amsterdam, the Netherlands), Erlangen (Germany), London (King’s College London, UK), Zurich (Switzerland), Valencia (Spain) and Vienna (Austria). The focus groups were audio recorded and the duration ranged from 30 to 96 min (mean: 63.5, SD: 25.4).

Four semi-structured interviews with governing level stakeholders per country were planned. These participants were recruited by consortium partners through email or telephone. They identified relevant representatives of this stakeholder group in their countries. Partners were asked to obtain the perspectives of different positions in the university hierarchy. There were no further standards set, as the partners best know the situation in their countries. However, they were asked to collect background information on different interviewee characteristics (position in the university, number of years of experience in the university setting) and to judge the level of influence/power regarding adoption, implementation processes and maintenance of online mental illness prevention.

Interviews were either conducted in person or over the phone by trained researchers in each country. Interviews were audio recorded and the duration ranged from 19 to 65 min (mean: 43.3, SD: 14.3). A minimum of 20–30 online questionnaires with DS were planned, per country.^21^ Student representatives, lecturers, university psychological services, researchers and student association groups were approached via email, which included a link to the questionnaire. Questionnaires included both quantitative and qualitative questions.

Focus group participants received monetary incentives ranging from 20 to 90 Euros. Participating stakeholders at governing level and DS did not receive any compensation.

### Sample description

In total, we conducted 14 focus groups with students (involving a total of 70 students) and 24 semi-structured interviews with stakeholders at a governing level. In each of the six countries, focus groups were conducted in one university town. Both students with and without an elevated risk for mental health disorders according to screening procedures participated. As universities are quite a unique setting, we reasoned that across different universities and participating European countries there would probably be more similarities than differences between the views of university stakeholders. Furthermore, different positions in the university hierarchy were approached, and therefore responses represent a range of ideas but also the opportunity to reach saturation of themes. Across all countries we obtained 149 online questionnaires with DS. Although we are unable to determine exact response rates amongst DS due to recruitment strategies (e.g. mass emails, where people were asked to forward the email on to colleagues) they do appear to be low for this group. For a detailed sample description see table 1.

**Table 1:** Characteristics of the sample, according to stakeholder group

University students: focus groups (Total *N* = 14)	
Number of focus groups per country, *n* (%)	
Austria	2 (14.3)
Switzerland	2 (14.3)
Germany	2 (14.3)
Spain	2 (14.3)
UK	4 (28.6)
Netherlands	2 (14.3)
Participants per gender, *n* (%)	
Females	50 (71.4)
Males	15 (21.4)
Unknown	5 (7.1)
**Staff at governing level: semi-structured interviews (total *N* = 24)**	
Number of interviews per country, *n* (%)	
Austria	4 (16.7)
Switzerland	4 (16.7)
Germany	4 (16.7)
Spain	4 (16.7)
Netherlands	4 (16.7)
UK	4 (16.7)
Type of interview, *n* (%)	
In-person	18 (75.0)
Telephone	6 (25.0)
Participants per gender, *n* (%)	
Females	10 (41.7)
Males	14 (58.3)
Stakeholder group,^a^ *n* (%)	
Governing sector	6 (17.6)
Care provider	12 (35.3)
Research	9 (26.5)
Education	5 (14.7)
Official representation of the target group	2 (5.9)
Level of influence,^b^ *n* (%)	
High	6 (25.0)
Moderate	4 (16.7)
Limited	13 (54.2)
Unknown	1 (4.2)
**Delivery staff: online questionnaire (Total *N* = 149)**	
Number of individuals per country, *n* (%)	
Austria	31 (20.8)
Switzerland	32 (21.5)
Germany	27 (18.1)
Spain	27 (18.1)
Netherlands	15 (10.1)
UK	17 (11.4)
Function, *n* (%)	
Student representative	23 (15.4)
Lecturer	73 (49.0)
Student association group	8 (5.4)
University psychological/counselling service	25 (16.8)
Researcher/PhD student	16 (10.7)
Other	4 (2.7)
Years of experiences in their function, mean (SD)/median	
Years	6.70 (7.74)/4.0

a Numbers do not add up due to stakeholders having multiple roles.

b Level of influence refers to the amount of influence on the adoption, implementation process and maintenance of online prevention in mental health.

### Data analysis

Focus groups and interviews were transcribed verbatim by researchers. The Dutch and Spanish transcripts and open-ended questions on the surveys were then translated into German or English. A thematic analysis ^23^ was conducted where the objective was to ascertain a wide range of themes rather than providing frequency of responses. The focus groups and interview transcripts as well as the answers on the open-ended questions of the online questionnaire were independently coded by two trained researchers in the Austrian team (S.K. and M.S.). Emerging themes and subcategories were organized in NVivo 11 Pro software. Afterwards the themes and subcategories were compared, and a categorical network was produced. As a final step, the results were merged and several members of the research team interpreted the final categorical network.^19^

There were no thematic differences between stakeholders from different countries. Thus, the results are not presented separately.

## Results

Identified themes and sub-themes from the interviews, focus groups and questionnaires are shown in Supplementary table S2 and illustrative quotes for each theme in Supplementary table S3. When no distinction has been reported, responses between stakeholders did not differ.

### Experiences

Most students and governing-level staff had no or limited experience with online prevention programmes. Although online interventions were mentioned, none were specifically aimed at prevention.

The majority (*N* = 109, 73%) of DS had either read or heard about online prevention programmes for mental health, and around half of the total DS sample (*N* = 71, 47.6%) had already looked into such programmes. Very few (*N* = 12, 8%) DS knew of any online prevention programmes for mental health available in their country. On a 10-point scale, from ‘no experience’ of online prevention programmes to ‘a lot of experience’, it was estimated that the level of experience for DS was low to medium (mean = 3.13, SD = 3.18).

### Underserved groups

The stakeholders were asked which student population(s) they would consider underserved regarding online prevention programmes. Besides students with psychological problems and subclinical symptoms of mental disorders (including a wide range of mental health problems), they also regarded students with sociodemographic risk factors (e.g. migration background, students with children and with full-time jobs) and students in specific stages of their studies (beginners but also doctoral students) as specific target groups that would benefit most from online prevention programmes.

### Attitudes

All stakeholders were asked about their attitudes towards online prevention programmes. Five advantages and three disadvantages were identified.

### Advantages

*Ease of access.* All stakeholders perceived that online prevention programmes reduced the physical and practical barriers to accessing care, in regards to time and geography. This allows users to flexibly engage in an intervention as they wish. It was thought that due to this ease of access, online prevention programmes are able to reach people on a larger scale in comparison to face-to-face methods (e.g. counselling). They were also seen as a better method to reach younger people, as the Internet is often integrated into their everyday life.

*For mental health prevention, these internet-based programmes would be useful, as students could use them in the evening, for example, or generally when they have time. (Governing level)*

*Help for sub-threshold mental health problems.* Online prevention programmes can provide help for people with sub-threshold symptoms who may not be able to seek face-to-face treatment. This was seen as a ‘first point of call’, helping people identify whether or not they need additional support.

*I'll take part in this programme and then I'll watch for myself, okay, if I'm honest with myself, I have a problem, yes or no, and then I can still say ‘Now I'll go to the doctor’. (Student)*

*Anonymity*. The online nature allows people to remain completely anonymous. Students, staff at governing level and DS said this could be preferable, as people could receive support without anyone else finding out, or without admitting you have a problem.

*Written format of delivery*. Having something written down was seen as beneficial for a number of reasons. Not only does it allow the user to recap on information, but students and staff at governing level also found it easier to process the information and self-reflect.

*…when I write this down, it's always a personal process that I can spend as much time as I want and then it's I'm, I think, even someone who likes to write a diary or something, because that's just then, you can reflect for yourself again (Student)*

*Destigmatization*. It was felt that online prevention programmes might help reduce stigma by making interventions widely available. They could also help people avoid stigmatization from others by making help-seeking more private. This was mentioned by all stakeholder groups, however was most prevalent amongst the student stakeholders.

*…So it's true that these interventions can maybe protect patients from a certain degree of shame, because when they come to us it is always easier to make contact over the Internet than turning up at the consulting room… (Governing level)*

### Disadvantages

*Lack of personal contact.* The lack of face-to-face contact was seen as a disadvantage and was mentioned by half of all stakeholders. This was because building a therapeutic relationship was seen an important factor in preventing and treating mental health problems. This was seen as a particular disadvantage for more severe problems, where face-to-face contact was seen as vital.

This lack of face-to-face contact was believed to result in more standardized approaches, aiming at a particular population (e.g. students). Stakeholders believed that this made it more difficult to individualize the approach. This might be problematic given different manifestations and causes of various mental disorders.

*Too impersonal. (Delivery staff)*

*Digital technology drawbacks*. All stakeholder groups raised data protection concerns in relation to online prevention programmes. Some student stakeholders felt unsure about how secure their information would be and would worry that ‘hackers could have access to it’.

In addition, digital technology was seen as overused among student populations. Online prevention programmes would increase this use, potentially further disconnecting them from the outside world.

*I do not think it's good to encourage that trend even more so that teens spend most of their time on the screen. You belong out there somehow. (Governing level)*

*Doubts about effectiveness of online programmes*. There are many different resources on the Internet which could result in uncertainty about which ones are effective. This came with the concern that any negative experience with an online prevention programme could potentially hinder the use of face-to-face treatment in the future due to the assumption that it will be the same. Doubts about the effectiveness of online prevention programmes were particularly prevalent for the stakeholders at governing level.

*I also had a friend in high school who had a lot of problems and her first step was to seek online help. And what they were telling her was not helpful at all, so she concluded well, therapy doesn’t help me, because the online interventions don’t give any good advices. (Student)*

DS were asked to weigh up advantages and disadvantages for online prevention programmes for mental health in comparison to face-to-face contact on a 10-point scale (−5 = much more disadvantages, 0 = neutral and +5 = much more advantages). Across all countries, the advantages outweighed the disadvantages (mean = 1.92, SD = 2.13). DS were also asked to what extent they would favour the integration of online interventions into their university, and if they would actively support this. This was rated on a 10-point scale (0 = not at all, 10 = absolutely). The mean ratings differed across countries, with DS in Spain having the most positive attitudes towards online prevention programmes (in favour of integrating: mean = 9.3, SD = 1.3; active support: mean = 9.4, SD = 1.5). DS in Austria had the least positive, but also most heterogeneous attitudes (in favour of integrating: mean = 6.71, SD = 3.1; active support: mean = 6.3; SD = 2.8).

### Needs—intervention topics, characteristics and aims

The stakeholders discussed which topics should be the focus for online prevention programmes for university students. Besides the prevention of mental disorders (e.g. anxiety disorders, depression and eating disorders), they also mentioned content on lifephase specific personal development (e.g. how to organise life, changes when entering university, life skills and love and sexuality) and academic challenges (e.g. learning and time management, test anxiety, stress and pressure and career start). DS rated the relevance of different predefined topics on a 10-point scale (0 = not at all relevant, 10 = very relevant), with stress disorders rated as most relevant (mean: 8.2).

When asked what an online intervention should include and look like, three main themes were identified.

*Basic conditions.* All stakeholders said that online interventions needed to be accessible. To enhance accessibility, students in particular mentioned that online programmes should be available on multiple devices (e.g. smartphones).

*…apps are really good in that everybody’s got a smart phone, and if it is designed as an app it’s easily accessible and you can probably do it in small chunks, rather than sitting down at a computer and kind of working through pages and pages of work (Student)*

*Tailoring of content.* All stakeholder groups felt it was important how any content was communicated to users, i.e. they felt it should be tailored to the target group by adapting it according to age and pre-existing psychological knowledge.

The layout of online programmes should be concise, modern and easy-to-use. Stakeholders said that to achieve this, PDF documents and Word Art should be avoided. Following a modular structure was felt to enhance conciseness and simplicity, making it more user-friendly.

*They should be appealing. They should trigger engagement. I think too much of these interventions are being offered in a format that makes it very easy to just ignore them. (Governing level)*

The content should be framed positively, focusing on the optimiz ation of health, rather than on mental health problems. For instance, stakeholders spoke about framing any such online prevention programme as being about ‘developing self-awareness’ or ‘helping you cope with student stress’. It was felt that this could help with destigmatization.

*Content.* The content itself should involve multimedia. Students said they do not want to read extensive information but including videos, pictures and audio clips would help make it more engaging. Videos, however, needed to be carefully selected as stakeholders said that poorly made videos could be off-putting.

*Ehm yeh, so I think it needs to be multimedia. I think a mix of video content, ehm, what I would call “e-nuggets”, sort of little learning bits that are interactive, ehm, and some written stuff. (Governing level)*

The programme content should allow for self-reflection. For example, questions should be asked about the content and videos, allowing the user to apply it to their own situation or experiences. Text boxes would be a helpful addition for reflection.

*It could be videos, and after watching you do a reflection with questions, or try to put yourself in the position of the person in the video so you can identify with them, interactive activities. (Student)*

Real life stories and personal experiences were also seen as important to students. This could be from previous students, celebrities and influential people who had struggled with mental health problems, and people who had previously completed a particular programme.

Finally, the content should be gamified and interactive. Students in particular spoke about having a ‘point system’ to allow users to monitor their progress and prevent people from ‘mindlessly flicking through the content’.

Regarding *aims*, all stakeholder groups discussed that online prevention programmes should not only aim at promoting mental health and preventing mental disorders but also at raising awareness and providing an insight to individuals mental health status by self-tests. Furthermore, in the long-term, the implementation of such programmes should also help to improve conditions at universities and academic success, e.g. by reducing dropout.

*One thing is to draw attention to possible problems, to possible help and motivate them to do something. (Governing level)*

### RE-AIM factors

All stakeholders were also asked about factors that would, in their opinion, foster or hinder reach, adoption, implementation and maintenance of online prevention programmes.

### Reach

When queried what factors would foster an online prevention programme reaching a large number of university students, two main themes were identified:

*Being made aware of the programme.* Online prevention programmes need to be embedded into a university setting to ensure people are aware of their availability. All stakeholder groups believed that sending ‘an email is not enough’, as students rarely read them. Instead, online programmes should be advertised on university websites, across different departments, at events and in lectures. This would require acceptance and support at many different levels across the university (from senior management to academic, administrative and health care staff). Social media was also thought to be a valuable way to make people aware, as it is often integrated into students’ lives. Continuous promotion across these platforms was seen as very important to ensure ongoing awareness amongst students of such initiatives/programmes.

*It must be enough visible everywhere. University event days, or poster on that, or teachers, whatever. And when you start studying, tutors or whatever you have in your programme, tell you about this thing. Not in an aggressive way, but just to let it, part of the university as many other things (Student)*

*Getting users to engage in the programme.* To persuade students to use an online prevention programme, it was felt to be important to provide them with information on the programme’s effectiveness. This could be done by linking information on prior research findings to the sign-up page. In addition, reminders were seen as important for engagement.

*Give examples about how it works. (Delivery staff)*

### Adoption

When queried about how to increase the number of universities who would be willing to initiate an online prevention programme, the following were identified:

*Resources.* A key factor that would influence universities, according to staff at governing level and DS, would be the amount of resources an online programme would require, such as the cost of running and maintaining the programme over time. Stakeholders believed that such costs should be met by the university rather than individual students. Human resources were also a consideration. Stakeholders at governing level spoke about how much time staff would need to invest and whether they would be willing to take on additional tasks relating to the implementation of online programmes.

*Resources (staff, time) available. (Delivery staff)*

*Attitudes.* There would need to be support across the whole university setting to help with adoption. A strong evidence base demonstrating the effectiveness of particular online programmes could help encourage this support. In addition, the attitudes of university health professionals were seen as important, as DS and staff at governing level believed that they would have more negative views due to beliefs that online programmes may replace their jobs.

*I get the impression that the, for a lot of counselling services like, they’re nervous about some of the online stuff because it sometimes, it’s sometimes pitched, or promoted as a replacement um, for face to face, um, and I suppose that’s more of a marketing thing. (Governing level)*

### Implementation

When stakeholders were asked which factors could foster or hinder whether prevention interventions could be delivered as intended, the following factors were identified:

*Usability.* An important factor for implementation was the usability and functionality of the programme, for instance, having a clear and easy-to-use design. The timing at which programmes were promoted to students was also an important consideration. Stakeholders said that the programme should not be introduced in the first couple of weeks of term or around exam period as it would be easily overlooked.

*I think it won’t be a problem I think if it’s well designed and easily accessible, I think if it’s robust, ehm, I think if it’s quite challenging but not challenging enough. (Governing level)*

*Responsibility of implementation.* Implementation was viewed as the universities’ responsibility. This could involve staff within the counselling services, university management staff or faculty staff.

### Maintenance

The extent to which an online intervention could become part of routine university practices and policies was queried. Two main themes were identified.

*Ongoing efforts to increase reach*. It was felt that online programmes could only be maintained if students continued to use them. To ensure this, any such programme needed to be accessible and regularly updated with new content.

*Structure*. It was felt important for any such programme to be embedded into the university structure and for a certain team or person to be responsible for monitoring the progression and success. Staff at governing level also identified having secure funding in place as an important factor.

*Yes, so/If we are realistic, the biggest factor is money. As long as the money is there, I think it's no problem at all to uphold that. I think that's the only (laughing) factor. So once it's implemented, it's super effective and relatively cost-effective, then there's no obstacle. The thing is, as long as the funding/If the funding is no longer there, then you can not pay more maintenance, if you do not pay any e-coaching or whatever. (Governing level)*

## Discussion

In one of the first European-based studies to look at university stakeholders’ attitudes and opinions on online mental health prevention for university students, several important results for planning and implementing programmes were identified. Overall, stakeholder groups had limited experience with specific online prevention programmes for students. Despite this, programme use was seen as acceptable. More was known by stakeholders about online treatment, as opposed to prevention, programmes. This is not surprising considering online prevention programmes are in their infancy,^10^^,^^24^^,^^25^ with the majority of previous literature investigating online treatment (e.g. Internet-based Cognitive Behavioural Therapy^17^^,^^19^).

Among several advantages identified for online prevention programmes, the biggest one was the low effort they require in staff time and infrastructure. Due to the lower effort and resources needed, such programmes may be accessible to a larger proportion of people, increasing how many people can benefit.^26^ The biggest disadvantage mentioned by student stakeholders was the lack of personal contact, which would make customization to the individual students’ needs more difficult. Previous research shows that students’ desire for human interaction is high when accessing help for their mental health,^20^ and its absence can influence the perceived efficacy of an online programme. In turn, perceived efficacy has been shown to be the most influential factor for students when making treatment choices,^16^ thus is also an important consideration in designing and promoting prevention programmes. Students indicate a preference towards blended interventions (a mix of online and face-to-face contact) over purely online treatment.^19^ Because online prevention programmes were perceived as standardized and too impersonal, this concern could be addressed by tailoring the programme content corresponding to the individual students’ needs according to online assessments and by integrating one-off face-to-face sessions, forums, self-monitoring tools and/or personalised feedback^27^ into the online platform.

Factors that influence the translation of online prevention and early intervention programmes into real-world situations were discussed (using the RE-AIM framework). Two predominant themes mentioned by stakeholders were the cost, and the need to embed the programmes into the university setting. Student mental health services are often overburdened and under financial pressure,^28^ so having a low-cost option is desirable. As cost is an important consideration, it is worth noting that online prevention programmes could cover a wide spectrum, with a variety of different associated costs. For instance, universities could opt for an ‘off the shelf’ generic programme which would be relatively cheap or design a more tailored university-specific one. Online prevention programmes could equip students with the relevant skills to prevent them from having to seek treatment later, easing the down-stream burden on services. Previous research has found that students lack knowledge about mental health services,^29^ so further integrating programmes into the university setting and routine services would be expected to have a positive impact. Saturating the university with awareness of such programmes would be one solution to increase knowledge and, by extension, engagement. However, a more promising possibility would be to offer online prevention of mental health in the framework of a health promoting university approach, a whole-systems approach which aims to integrate health promotion into the university policies, culture and processes.^14^^,^^15^^,^^30^ *‘… so it’s not just a kind of an add-on or something that’s an afterthought, this is something the university is very committed to…’* (interview quote, governing level).

Strengths of this study include a large and diverse sample of stakeholders across six European countries which allowed us to capture a range of representative opinions from students, governance and DS. Findings augment the existing literature by considering the RE-AIM framework in designing future dissemination and implementation of programmes. Discrepancies have been identified about the perceived helpfulness of online interventions and the actual use,^31^ so understanding RE-AIM factors provides valuable information with regard to use beyond research.

This study has some limitations which need to be considered. A convenience sample was used, which may have introduced a bias in responses. For example, people who favour the integration of technology into mental health care might be more likely to participate. Secondly, the majority of focus group participants (71.4%) were female which may have lessened thematic saturation. Response rates to the online questionnaire were low in all countries which may limit the representativeness of findings. Additionally, due to the limited knowledge of prevention programmes amongst all stakeholders, the discussions tended to focus largely on online programmes in general. We therefore, cannot be certain that these views and opinions apply when specifically considering online prevention programmes.

This study provides valuable information about the values and needs of different stakeholders with regards to online mental health prevention programmes in university settings. Aspects that should be considered when designing and implementing future prevention programmes for university students are interactivity to increase attraction (e.g. through gamification) and personalization to increase relevance and adherence. Students should also be involved in the development stage as doing so could inform engagement and potentially reduce the high dropout rates seen in previous studies.^12^^,^^14^^,^^16^^,^^18^ Future research should, however, focus on differentiating treatment and prevention interventions to examine potential differences in experience and attitudes. It would be helpful to understand how best to engage students or at least to target those at high risk rather than those with current mental health problems, as they are potentially harder to reach. It is also important to investigate the effectiveness of online prevention programmes prior to disseminating them widely.

## Supplementary data

Supplementary data are available at *EURPUB* online.

## Supplementary Material

ckab040_Suppelementary_Material
