# Supplementary material for: Online prevention programmes for university students: stakeholder perspectives from six European countries
Source: Eur J Public Health. 2021 Jul 7;31(Suppl 1):i64–70. doi: 10.1093/eurpub/ckab040 (PMC8495721; doi:10.1093/eurpub/ckab040)
Supplement: ckab040_Suppelementary_Material [file ckab040_suppelementary_material.pdf]

Supplementary material to

Online prevention programmes for university students: stakeholder perspectives from six European countries

Irish, M., Kuso, S., Simek, M., et al.

Corresponding author:

Madeleine Irish
King's College London, Institute of Psychiatry, Psychology and Neuroscience, Box P059, De Crespigny Park, London SE5 8AF, UK
Phone: 0207 848 0246
Email: madeleine.irish@kcl.ac.uk

Table 2. Summary of themes across focus groups, interviews, and surveys.

**Research question**	**Predefined themes (framing questions)**	**Derived Themes**	**Sub-theme**
Experiences	Experiences		
Underserved groups	Underserved groups		
Attitudes	Advantages		Ease of access
			Help for sub-threshold mental health problems
			Anonymity
			Written format of delivery
			Destigmatisation
	Disadvantages		Lack of personal contact
			Digital technology drawbacks
			Doubts about the effectiveness of online programmes
Needs	Topics		
	Characteristics	Basic conditions	Accessibility
		Tailoring of content	Adequate to target group
			Attractiveness
			Positive framing

		Content	Multimedia
			Self-reflecting content
			Real-life stories
			Gamifications and interactivity
	Aims		
Reach	Reach	Being made aware of the programme	Embedding the programme into the university setting
			Acceptance and support
			Social media
		Getting users to engage in the programme	Effectiveness
			Reminders
Adoption	Adoption	Resources	Cost
			Staff requirements
		Attitudes	
Implementation	Implementation	Good usability and function	
		Responsibility	
Maintenance	Maintenance	Ongoing efforts to increase reach	
		Structure	Allocated responsibility
			Secured finance

Table 3. Summary of illustrative quotes and answers from focus groups, interviews, and surveys, according to themes identified.

Subtheme	Quotes (stakeholder)
**Advantages**	
**Ease of access**	*"For mental health prevention, these internet-based programmes would be useful, as students could use them in the evening, for example, or generally when they have time." (Governing level)*
	"I always have my phone with me, I have my laptop and if I want to access the programme, then I do not have to look for an address, I do not have to go anywhere. I have it with me and I can do it right away, without spending energy, time and money on it." (Student)
	"Reaching out to more people. A therapist can hold a maximum of five sessions a day, but using these tools could reach out to a hundred people or more." (Student)
	"Flexible and accessible from everywhere." (Delivery staff)
**Help for sub-threshold mental health problems**	*"I also think this kind of stuff is an excellent supplementary resource, and it can help students to highlight where to go to next, so it's not REPLACING therapy, but it's certainly making people aware 'now I'm probably at the point where I probably need something" (Governing level)*
	"I'll take part in this programme and then I'll watch for myself, okay, if I'm honest with myself, I have a problem, yes or no, and then I can still say 'Now I'll go to the doctor'". (Student)
	"Low threshold." (Delivery staff)
**Anonymity**	*"Because maybe you really want to operate the programme, but you don't want other people to know that you're doing it, because you may feel ashamed or you don't want to share your own thoughts with other people" (Student)*
**Written format of delivery**	*"I also think having something in writing, where there is an opportunity for repetition and going back, is invaluable." (Governing level)*
	"…when I write this down, it's always a personal process that I can spend as much time as I want and then it's I'm, I think, even someone who likes to write a diary or something, because that's just then, you can reflect for yourself again" (Student)
	"At your own pace." (Delivery staff)
**Destigmatisation**	*"…So it's true that these interventions can maybe protect patients from a certain degree of shame, because when they come to us it is always easier to make contact over the Internet than turning up at the consulting room…" (Governing level)*
	"…maybe the idea of normalise the process of mental health. So it's something that you are going to have in your daily life." (Student)

**Disadvantages**	
**Lack of personal contact**	*"The therapeutic bond is vital and I think that these technologies or online treatments are a very good tool, but without neglecting the human side." (Student)*
	"…the therapeutic relationship also represents one of the major factors of impact. And also, I think, for extremely unstable people, that's just what gives the most support, I think. Independent of techniques." (Governing level)
	"But online for me it is just for smaller issues, for not so serious problems, but as soon as that really, where I realise, that is now somehow very difficult or so, then I would not use this online, because I then also, if I, because then I would really like the honest opinion or so for example and then I want to see someone who sits opposite me…" (Student)
	"It just has to be standardised so to speak and that is certainly a disadvantage in a way, that it is not created individually for you." (Student)
	"Too impersonal." (Delivery staff)
**Digital technology drawbacks**	*"I think that privacy might be both a disadvantage and an advantage, it probably depends whether you trust the website so if it seems maybe a bit dodgy I would be really scared that it's actually like someone like taking information about my mental health. Like you can't be sure that it's protected." (Student)*
	"I do not think it's good to encourage that trend even more so that teens spend most of their time on the screen. You belong out there somehow." (Governing level)
**Doubts about the effectiveness of online programmes**	*"I would know there are many websites, so you never know which is the reliable one and which is not. So yeah, again, you don't have this knowledge. And sometimes it might have benefits for you, but sometimes it might have exact the opposite effect." (Student)*
	"I also had a friend in high school who had a lot of problems and her first step was to seek online help. And what they were telling her was not helpful at all, so she concluded well, therapy doesn't help me, because the online interventions don't give any good advices." (Student)
	"Information could be misunderstood." (Delivery staff)
**Characteristics**	
**Basic conditions**	*"…apps are really good in that everybody's got a smart phone, and if it is designed as an app it's easily accessible and you can probably do it in small chunks, rather than sitting down at a computer and kind of working through pages and pages of work" (Student)*
**Tailoring of content**	*"And also something we've been thinking about is the amount of psychological knowledge that you assume, so whether you assume that people are quite well up on this sort of stuff, or whether they have a very low-level knowledge, it's kind of a tricky balance to strike." (Student)*
	"The programmes should definitely be attractive and interactive. This means that not only pdfs should be uploaded. " (Governing level)

	"They should be appealing. They should trigger engagement. I think too much of these interventions are being offered in a format that makes it very easy to just ignore them." (Governing level)
	I1: "Yeh, so if you kind of framed it more as a tool for learning about yourself."<br><br>*P3: "Yeh, I think that'd be good, because there's still a bit of iffy-ness around admitting that you have a problem as well, not everyone would so readily say that, so I think if you marketed it that way, got an angle on that, useful for you to learn a bit more about yourself and how you deal with different situations, different stresses, I think people would be inclined to take a look at that." (Student)*
**Content**	*"Maybe like, like instead of just having loads of information, maybe like a short video or something. Like a Youtube video or something, that takes like a couple of minutes but explains everything that it needs to say cause, (laughs) people our age don't like reading much, we prefer to watch a video and be told about it. Um, but yeah good points." (Student)*
	"Ehm yeh, so I think it needs to be multimedia. I think a mix of video content, ehm, what I would call "e-nuggets", sort of little learning bits that are interactive, ehm, and some written stuff." (Governing level)
	"It could be videos, and after watching you do a reflection with questions, or try to put yourself in the position of the person in the video so you can identify with them, interactive activities." (Student)
	"Maybe also some videos of people who talk about their experiences. Maybe PhD students who are a little bit older, students who talk about their years in university and what they experienced, what helped them, so you have like a specific example of a person that shares their own things." (Student)
	"And I also think like small reminders every day on your phone, because most of the time we feel bad. But we forget we recognise that we feel bad. We're like overwhelmed in this situation, so it's better, like having small reminders today. Like take a breath, or go out for a walk, how do you feel?" (Student)
**Aims**	*"I think the most important thing is to create awareness, because if someone is not aware of the issues, that person is not going to go to prevention, because he doesn't know what it is going to be for." (Student)*
	"Yeah, like a quick set of questions or something, and then someone's like 'alright this is fine', like maybe what's wrong with you – well, not wrong, but like, these are your issues, that you might like to address." (Student)
	„One thing is to draw attention to possible problems, to possible help and motivate them to do something." (Governing level)
**REAIM**	

**Reach**	*"And I think well, you have to be much more innovative and much closer to the target population who you would like to address. Let's say if we do it between twenty and thirty, well, social media should be involved" (Governing level)*
	"It must be enough visible everywhere. University event days, or poster on that, or teachers, whatever. And when you start studying, tutors or whatever you have in your programme, tell you about this thing. Not in an aggressive way, but just to let it, part of the university as many other things" (Student)
	"Being accepted by health professionals, something, ehm, that's widely promoted across services actually, not just in the counselling service. I think also if we can get academic, ehm, departments, you know, if they know about it, so I guess you know, awareness, raising awareness, will increase that." (Student)
	"I think there should necessarily, if there is such a website, efficacy studies that have been conducted in the context, be linked on this page" (Student)
	"Maybe a programme with a reminder on the phone might not be bad. I mean today we are connected to everything anyway and if you are working with the PC and you are writing some work and then "BLING; Do you want to talk about it again? "or something." (Student)
	"Give examples about how it works." (Delivery staff)
**Adoption**	*"One important factor is, of course, the financing, which must be secured, possibly also sustainably secured, i. E. over several years and not just for a year-long research project. If sustainable funding can be guaranteed, this would certainly encourage adoption or, conversely, impede adoption if that is not the case." (Governing level)*
	"Yeah, well it's just the question how willing are people to take on an extra task. So that's a critical point to keep in mind." (Governing level)
	"Ehm, on a global scale I suppose, it would require the institution again to both believe and have confidence in this as being a viable option, so it's not just kind of an add-on or something that's an afterthought, this is something the university is very committed to and believes in, ehm, and then I think, if it worked well, it would trickle down on almost all levels…" (Governing level)
	"I get the impression that the, for a lot of counselling services like, they're nervous about some of the online stuff because it sometimes, it's sometimes pitched, or promoted as a replacement um, for face to face, um, and I suppose that's more of a marketing thing." (Governing level)
	"Resources (staff, time) available." (Delivery staff)
**Implementation**	*"I think it won't be a problem I think if it's well designed and easily accessible, I think if it's robust, ehm, I think if it's quite challenging but not challenging enough." (Governing level)*
	"Maybe I would be willing to pay something, but I still think it's the job of the university." (Student)

	"I think, in the case of (university name) it has to fall within, I would say it's a very senior student services, ehm, slash senior counselling-type, because of that mental health side of things…" (Governing level)
	"Cooperation of students and administration." (Delivery staff)
**Maintenance**	*"I think, there's either a, in order for it to truly be embedded there needs to be expertise and ownership within the university for looking after it." (Student)*
	P4: "So if lecturers might point out that again and again." (Student)<br><br>*P3: "I think it's just important that the people who pass this on, such as professors, that they really pass that on as something important." (Student)*
	"Yes, so / If we are realistic, the biggest factor is money. As long as the money is there, I think it's no problem at all to uphold that. I think that's the only (laughing) factor. So once it's implemented, it's super effective and relatively cost-effective, then there's no obstacle. The thing is, as long as the funding / If the funding is no longer there, then you can not pay more maintenance, if you do not pay any e-coaching or whatever." (Governing level)

I CARE

GA 634757

I CARE

Instruments

Stakeholder Survey Instruments

(Internet-based prevention in the field of mental health)

This project has received funding from the European Union's Horizon 2020 research and innovation programme under grant agreement No. 634757.

Table of Contents

1 Focus group topic guide (Target groups or Beneficiaries) 3

2 Semi-structured Interview Guide (Policy makers) 6

3 Online-Questionnaire (Facilitators or Enablers) 10

1 Focus group topic guide (Target groups or Beneficiaries)

Introduction

Welcome to our session. Thanks for taking the time to join us to talk about prevention in the field of mental health. My name is ….. and assisting me is …... We're researchers at the ….

At the moment we are working on a project, which is funded by the European Commission. The University of Dresden is leading the research project and researchers from 13 other research institutions and universities across Europe are participating. The aim of this project is to implement Internet-based interventions that aim to prevent mental health problems and disorders in different settings, such as universities, schools and the general health care system, across Europe. In order to successfully develop and implement these programs we would first like to explore the needs, values, attitudes and experiences of different stakeholder groups.

Thus, your views will provide valuable input for our programmes, so your opinion is very important to us. There are no wrong answers but rather differing points of view. Please feel free to share your point of view even if it differs from what others have said. Keep in mind that we are just as interested in negative comments as positive comments.

We are tape recording the session because we don't want to miss any of your comments. People often say very helpful things in these discussions and we can't write fast enough to get them all down. We won't use any names in our reports. You may be assured of complete confidentiality.

Before we can start we would need you to sign the consent form. Please read and sign it and let us know if you have any questions.

KEY TOPICS *[Add probes for fuller response and/or change order depending on situation and participants]*

Warm up (refers to "needs")

1. What do you think are the biggest mental health challenges for the health system in your country / for students within the first year at university / for pupils between 14 and 19?

[Relevance of topic "prevention in the field of mental health" in general]

2. How do you feel about prevention in the field of mental health in schools/universities/ the health care system in general?

[Experiences with Internet-based interventions]

3. What are your experiences with Internet-based interventions that aim to prevent mental health problems and disorders?
   a) Which Internet-based interventions that aim to prevent mental health problems and disorders are available for pupils at your school / students at your university/ in the health care system in your country? [Probe: name, age group, focus?]

[Values and attitudes]

4. What do you think about offering Internet-based interventions that aim to prevent mental health problems and disorders within the health care system in your country /in universities/in schools?
   a) What do you consider as the most important advantages of Internet-based interventions that aim to prevent mental health problems and disorders compared to face-to-face interventions?
   b) What do you consider as the most important disadvantages of Internet-based interventions that aim to prevent mental health problems and disorders compared to face-to-face interventions?

[Needs]

5. Which topics need to be focused on when offering Internet-based interventions that aim to prevent mental health problems and disorders in the health care system in your country /in universities/in schools?
   a) Which overall aims need to be focused on?
   b) Which characteristics would you consider important for Internet-based interventions that aim to prevent mental health problems and disorders in schools/universities/ the health care system in your country?
   c) Which groups of pupils / university students / individuals would you consider most underserved regarding Internet-based interventions that aim to prevent mental health problems and disorders?

*[Barriers and facilitators for reach, adoption, implementation, maintenance]**

[REACH]

6. Which factors need to be considered to make Internet-based interventions attractive (for pupils/ students)?

[IMPLEMENTATION]

7. Which factors need to be considered in order to successfully integrate Internet-based interventions that aim to prevent mental health problems and disorders in the daily (school/university) routine?

[CONCLUSION/ END]

8. Is there anything else you would like to tell us?

Thank the participants for their time. Reiterate that the discussions will remain confidential. Tell them that they are welcome to contact the research team to ask questions at a later date if they wish.

**level of adoption is only relevant on an organizational level, "maintenance" is covered by implementation; asking about "maintenance" and "future research" does not seem adequate at this early stage of the project.*

2 Semi-structured Interview Guide (Policy makers)

The following information will be pre-filled by the interviewer and will not be asked directly from the respondent:

1. Indicate Stakeholder Group (e.g. funders, government, health insurances, etc.)

2. Indicate level of influence (limited, medium, high)

3. Indicate country where currently based

4. Indicate type of interview (in-person vs. telephone)

Introduction

Thanks for taking the time to join us to talk about prevention in the field of mental health. My name is ….. (if applicable: and assisting me is ……) I am a researcher at the ….

At the moment we are working on a project, which is funded by the European Commission. The University of Dresden is leading the research project and researchers from 13 other research institutions and universities across Europe are participating. The aim of this project is to implement Internet-based interventions that prevent mental health problems and disorders in different settings, such as universities, schools and the health care system in general, across Europe. In order to successfully develop and implement these programs we would first like to explore the needs, values, attitudes and experiences of different stakeholder groups.

Your views will provide valuable input for our programmes, so your opinion is very important to us. Also, please keep in mind that we are just as interested in negative comments as positive comments.

We are tape recording the session because we don't want to miss any of your comments and we can't write fast enough to get them all down. We won't use any names in our reports. You may be assured of complete confidentiality.

Before we can start we would need you to sign the consent form. Please read and sign it and let us know if you have any questions.

***KEY TOPICS** [Add probes for fuller response and/or change order depending on situation and interviewee]*

Warm up

1. Can you tell us a bit about your own work (if applicable: that focuses on mental health)?
   - What is the specific focus of your work (e.g. programme implementation or evaluation, policy)?
   - Can you give some examples of your work?
2. From your own experience in this sector, what would you say are the biggest mental health challenges for the health care system in your country / for students within the first year at university / for pupils between 14 and 19?

[Relevance of topic "prevention in the field of mental health" in general]

3. How do you feel about prevention in the field of mental health in schools/universities/ the health care system in general?
   - How relevant are interventions in this field?
   - Who should be responsible for initiatives regarding prevention in the field of mental health in the health care system / schools / universities?

[Underserved populations]

4. Which groups of pupils / university students / individuals would you consider most underserved regarding prevention of mental health problems and disorders?
   - How can those groups be reached?

[Experiences with Internet-based interventions]

5. What are your experiences with Internet-based interventions that aim to prevent mental health problems and disorders?
   - Which Internet-based interventions that prevent mental health problems and disorders are available for pupils at your school / university students at your university/ within the health care system in your country? [name, age group, focus]

[Needs]

6. Which topics need to be focused on when offering Internet-based interventions that aim to prevent mental health problems and disorders in schools/universities/ the health care system in your country?
   - Which overall aims need to be focused on?
   - Which characteristics would you consider important for Internet-based interventions that aim to prevent mental health problems and disorders in schools/universities/ the health care system in your country?

- Which groups of pupils / university students / individuals would you consider most underserved regarding Internet-based interventions that aim to prevent mental health problems and disorders?

[Values and attitudes]

7. What do you think about offering Internet-based interventions that aim to prevent mental health problems and disorders in schools/universities/ the health care system in your country?
   - What do you consider as the most important advantages of Internet-based interventions that aim to prevent mental health problems and disorders compared to face-to-face interventions?
   - What do you consider as the most important disadvantages of Internet-based interventions that aim to prevent mental health problems and disorders compared to face-to-face interventions?
8. Would you actively support the integration of Internet-based interventions that aim to prevent mental health problems and disorders in your school / your university / the health care system in your country?

[Barriers and facilitators for reach, adoption, implementation, and maintenance]

The following questions focus on potential **hindering and fostering context factors regarding different program phases**. In the research model that we are using, these phases are described as “reach, adoption, implementation and maintenance”. In the following part we give you a short explanation about these phases and after that we ask you about potential barriers and facilitators that could apply to each of these phases.

[REACH]

<u>Definition:</u> **REACH** means the absolute number, proportion, and representativeness of individuals who are willing to participate in a given initiative.

9. Which factors could <u>foster</u> REACH of pupils / university students / individuals regarding the use of an Internet-based intervention program that aims to prevent mental health problems and disorders?
10. Which factors could <u>hinder</u> REACH of pupils / university students / individuals regarding the use of an Internet-based intervention program that aims to prevent mental health problems and disorders?

[ADOPTION]

<u>Definition:</u> **ADOPTION** means the absolute number, proportion, and representativeness of settings and intervention agents who are willing to initiate a program.

11. Which factors could <u>foster</u> the ADOPTION of Internet-based intervention programs that aim to prevent mental health problems and disorders in schools / universities / the health care system?

12. Which factors could hinder the ADOPTION of Internet-based intervention programs that aim to prevent mental health problems and disorders in schools / universities / the health care system?

[IMPLEMENTATION]

Definition: At the setting level, **IMPLEMENTATION** refers to the intervention agents' fidelity to the various elements of an intervention's protocol. This includes consistency of delivery as intended and the time and cost of the intervention.

13. Which factors could foster the IMPLEMENTATION of Internet-based intervention programs that aim to prevent mental health problems and disorders in schools / universities / the health care system?
14. Which factors could hinder the IMPLEMENTATION of Internet-based intervention programs that aim to prevent mental health problems and disorders in schools / universities / the health care system?
15. Who should be responsible for the IMPLEMENTATION of Internet-based interventions that aim to prevent mental health problems and disorders in schools / universities / the health care system?

[MAINTAINANCE]

Definition: **MAINTAINANCE** means the extent to which a program or policy becomes institutionalized or part of the routine organizational practices and policies. **MAINTAINANCE** is also known as sustainability.

16. Which fostering factors need to be considered that Internet-based interventions that aim to prevent mental health problems and disorders can be integrated in the regular practice of schools / universities / the health care system?
17. Which hindering factors need to be considered that Internet-based interventions that aim to prevent mental health problems and disorders can be integrated in the regular practice of schools / universities / the health care system?

[FUTURE RESEARCH]

18. What would you suggest for future research regarding interventions that prevent mental health problems and disorders in general?

[CONCLUSION/ END]

19. Is there anything else you would like to tell us?

Thank the participant for their time. Reiterate that the interview will remain confidential. Tell them that they are welcome to contact the research team to ask questions at a later date if they wish.

3 Online-Questionnaire (Facilitators or Enablers)

[INTRODUCTION]

Dear Participant,

ICare (Integrating Technology into Mental Health Care Delivery in Europe) is a project, which is funded by the European Commission. The University of Dresden is leading the research project and researchers from 13 other research institutions and universities across Europe are participating. The aim of this project is to implement Internet-based interventions that prevent mental health problems in different settings, such as universities, schools and the health care system in general, across Europe.

In order to successfully develop and implement these programs we would primarily like to explore the needs, values, attitudes and experiences of different stakeholder groups.

Your views will provide valuable input for our programs, so your opinion is very important to us.

This survey consists of 27 questions and will take about 30 minutes to complete. This survey is completely anonymous, we will not ask you to provide any information concerning your person and the organization you are working in.

Thank you for participating in this stakeholder survey!

Coordination:

TECHNISCHE UNIVERSITÄT DRESDEN
EPC European Project Center

Partners:

MEDICAL UNIVERSITY OF VIENNA
FERDINAND PORSCHE FERN FH
Universität Zürich UZH
ISGF
VU UNIVERSITY AMSTERDAM
KING'S College LONDON University of London
LSE
WESTFÄLISCHE WILHELMS-UNIVERSITÄT MÜNSTER
VNIVERSITAT DE VALÈNCIA
UNIVERSITAT JAUME I
FAU FRIEDRICH-ALEXANDER UNIVERSITÄT ERLANGEN-NÜRNBERG
UNIVERSITÄT BERN

Advisory Committee:

STANFORD SCHOOL OF MEDICINE
Dachverband
THE UNIVERSITY OF MEMPHIS

This project has received funding from the European Union's Horizon 2020 research and innovation program under grant agreement No 634757.

[CHOOSE COUNTRY]

	**Please choose your country!**<br>**Bitte geben Sie Ihr Land an!**<br>**Por favor, elija su país!**<br>**Kies uw land!**
○	United Kingdom
○	Deutschland
○	Österreich
○	Schweiz
○	Nederland
○	Espana

[IF United Kingdom → English language version
IF Deutschland OR Österreich OR Schweiz → German language version
IF Nederland → Dutch language version
IF Espana → Spanish language version]

[FUNCTION]

**1.**	**Please indicate the setting of which you currently represent/ in which you are working at the moment** (If you represent/work (in) several settings, please choose the setting, (in) which you represent/work most of your time.)
○	**Health Care System** (health care professionals, e.g. general practitioners, psychiatrists, pediatricians, psychologists, psychotherapists, nurses)
○	**School System** (e.g. school teachers, school authorities, school psychological services, school counselling services, student representatives)
○	**University System** (e.g. university teachers , university authorities, university psychological services, university counselling services, university students association and groups, student representatives)

[IF Health Care System → Health Care System version
IF School → Version for School Setting
IF University → Version for University Setting]

1a.	**Please indicate your role** (If you have more than one role, please choose the role that you fulfil most of the time.)
○	Pupils' representative
○	School teacher
○	School principal
○	School psychologist
○	School physician
○	School social worker
○	Other function within the school setting
○	University student representative
○	University teacher / lecturer
○	Member of the university student association / group
○	University psychological / counselling service
○	Other function within the university setting
○	General practitioner
○	Pediatrician
○	Psychologist
○	Psychotherapist
○	Social Worker / Social Education Worker
○	Psychiatrist
○	Nurse
○	Other function within the health care system

1b.	**Please indicate the number of years of experience you have in the role chosen above.**

Numeric open answer

[IF "Other function within the school setting" in Question 1]:

**1c.** **Which other role(s) within the school setting do you have?**
Open answer

[IF "Other function within the university setting" in Question 1]:

**1d.** **Which other role(s) within the university setting do you have?**
Open answer

[IF "Other function within the health care system" in Question 1]:

**1e.** **Which other role(s) within the health care system do you have?**
Open answer

[A. Relevance of topic "prevention in the field of mental health" in general]

This questionnaire deals with prevention in the field of mental health. First of all, please read through what we understand by the term "prevention": By prevention, we mean all measures that are taken

- **Prior to the occurrence of mental health problems or**
- **When first symptoms of mental health problems occur**

In other words:
We are dealing with measures that help…

- **To prevent mental health problems or**
- **Prevent the progression of first symptoms.**

In the following, please think about such preventive measures for pupils/university students/ individuals in your country.

The next questions deal with your views on prevention in the field of mental health in general. By this, we mean all measures that are taken prior to the occurrence of mental health problems or when first symptoms occur.

**2.**	**To what extent do you personally consider prevention in the field of mental health relevant in for pupils in schools/ for students in universities/ the health care system in your country?**
Slider	not at all relevant — 0 1 2 3 4 5 6 7 8 9 10 — very relevant

**3.**	**To what extent do you consider your school / your university / the health care system responsible for prevention in the field of mental health for pupils / university students?**
Slider	not at all responsible — 0 1 2 3 4 5 6 7 8 9 10 — very responsible

**4.**	**To what extent do you in your role feel personally responsible for prevention initiatives for mental health for pupils / for students / in the health care system in your country?**
Slider	not at all responsible — 0 1 2 3 4 5 6 7 8 9 10 — very responsible

[B. Underserved populations]

**5.**	**On your opinion, which group(s) of pupils / university students / individuals might most benefit from prevention initiatives in the field of mental health?**
Group 1	*Open answer*
Group 2	*Open answer*
Group 3	*Open answer*

**5a.**	**How can those group(s) that you mentioned be best reached by prevention measures in the field of mental health in schools / universities / health care system?** *(Please give a short suggestion for each of the groups)*
[Fill in answer of Group 1 of Q5 here]	*Open answer*

[Fill in answer of Group 2 of Q5 here]	*Open answer*
[Fill in answer of Group 3 of Q5 here]	*Open answer*

The following part deals with Internet-based prevention programs in the field of mental health.

In contrast to conventional approaches, participants receive interactive and tailored information and exercises related to mental health via computer or mobile devices (smartphone, tablet). The interaction with coaches, moderators or experts as well as with other participants usually doesn't take place in person but via emails, online messages or discussion boards.

Used continuously over a certain period of time, Internet-based programs can contribute preventing mental health problems or progression of first symptoms.

Internet-based prevention programs are also called computer-based, web-based or online programs.

The following questions deal with your experiences, needs and attitudes regarding **Internet-based prevention programs** in the field of mental health.

[C. Experiences with Internet-based interventions]

**6.**	**Please state, if the following statements regarding Internet-based prevention programs in the field of mental health apply to you:**
○ ○ Yes No	I have read or heard about it.
○ ○ Yes No	I have had a look at such programs.
○ ○ Yes No	I have used such programs myself (to prevent own mental health problems).
○ ○ Yes No	I have implemented or helped to implement such programs (at a school, university, in the health care system or in a different setting).
○ ○ Yes No	I have counselled individuals using such programs as a coach, moderator, or an expert.

7.	**Are there any Internet-based prevention programs in the field of mental health available for pupils at your school / students at your university / individuals in the health care system in your country?**
○	*Yes*
○	*No*
○	*I'm not sure / I don't know*

[IF "YES "on question 7→ proceed with question 7a
IF "NO" or "I'm not sure" on question 7→ proceed with question 8]

7a. Please provide more details about these programs in the table below:

**Name of the program** (if available)	**Age group** Please provide the age group the program is targeted to; write "general" if the program is not targeted at a specific age	**Focus** Please state the focus of the program – e.g. depression, anxiety, eating problems; if there is no specific focus: write "general"	**Aim(s) of the program** please describe in short the aim(s) of the program, e.g. "enhancing self-esteem"
Open answer	*Open answer*	*Open answer*	*Open answer*
Open answer	*Open answer*	*Open answer*	*Open answer*
Open answer	*Open answer*	*Open answer*	*Open answer*
Open answer	*Open answer*	*Open answer*	*Open answer*
Open answer	*Open answer*	*Open answer*	*Open answer*

8.	**Altogether, <u>how much experience</u> do you have with Internet-based prevention programs in the field of mental health?**

Slider	No experience whatsoever 0 1 2 3 4 5 6 7 8 9 10 Very much experience

[D. Needs]

The next set of questions deal with topics, aspects and characteristics for Internet-based prevention programs in the field of mental health addressed to pupils at schools / students at universities / in the health care system in your country.

**9.**	**How relevant do you consider the following <u>topics</u> for Internet-based prevention programs in the field of mental health?**
General mental health problems (no specific focus)	not at all relevant 0 1 2 3 4 5 6 7 8 9 10 very relevant
Anxiety Disorders	not at all relevant 0 1 2 3 4 5 6 7 8 9 10 very relevant
Depressive Disorders	not at all relevant 0 1 2 3 4 5 6 7 8 9 10 very relevant
Eating Disorders	not at all relevant 0 1 2 3 4 5 6 7 8 9 10 very relevant
Personality Disorders (perception and behavior are not flexible and appropriate; e.g. excessive distrust, violation of social norms, persistent worries, etc.)	not at all relevant 0 1 2 3 4 5 6 7 8 9 10 very relevant
Substance Use Disorders (e.g. abuse of alcohol, drugs, nicotine)	not at all relevant 0 1 2 3 4 5 6 7 8 9 10 very relevant
Stress Disorders	not at all relevant 0 1 2 3 4 5 6 7 8 9 10 very relevant
Mental and physical health Promotion	not at all relevant 0 1 2 3 4 5 6 7 8 9 10 very relevant

Resilience(ability to cope with crises)	not at all relevant 0 1 2 3 4 5 6 7 8 9 10 very relevant
Self-esteem	not at all relevant 0 1 2 3 4 5 6 7 8 9 10 very relevant

**9a.**	**Which other topics would you consider very relevant?**
Open answer	

**10.**	**How relevant do you consider the following aspects for Internet-based prevention programs in the field of mental health addressed to pupils in schools / students in universities / in the health care system in your country?**
Prevent onset of mental health problems and disorders	not at all relevant 0 1 2 3 4 5 6 7 8 9 10 very relevant
Reduce risk factors for mental problems and health disorders	not at all relevant 0 1 2 3 4 5 6 7 8 9 10 very relevant
Increase protective factors	not at all relevant 0 1 2 3 4 5 6 7 8 9 10 very relevant
Prevent progression of first symptoms of mental disorders	not at all relevant 0 1 2 3 4 5 6 7 8 9 10 very relevant
Reduce severity of first symptoms of mental disorders	not at all relevant 0 1 2 3 4 5 6 7 8 9 10 very relevant

**11.**	**How relevant do you consider the following characteristics for such prevention programmes?**
Usability (e.g. easy to use)	not at all relevant 0 1 2 3 4 5 6 7 8 9 10 very relevant
Attractiveness (e.g. modern, appealing interface)	not at all relevant 0 1 2 3 4 5 6 7 8 9 10 very relevant

User Empowerment (competence for self-help)	not at all relevant 0 1 2 3 4 5 6 7 8 9 10 very relevant
Recognizability of the benefit for the user	not at all relevant 0 1 2 3 4 5 6 7 8 9 10 very relevant
Anonymity	not at all relevant 0 1 2 3 4 5 6 7 8 9 10 very relevant
Avoidance of stigmatization (reducing feelings of being prejudged and marginalized)	not at all relevant 0 1 2 3 4 5 6 7 8 9 10 very relevant
Motivational Elements (e.g. feedback, rewards)	not at all relevant 0 1 2 3 4 5 6 7 8 9 10 very relevant
Ease of access	not at all relevant 0 1 2 3 4 5 6 7 8 9 10 very relevant
Economic Factors (costs, time exposure)	not at all relevant 0 1 2 3 4 5 6 7 8 9 10 very relevant
Reminders(e.g. via SMS or email) to participate regularly until the end	not at all relevant 0 1 2 3 4 5 6 7 8 9 10 very relevant
Dynamic and Personalized Feedback (e.g. suggestions related to entered data)	not at all relevant 0 1 2 3 4 5 6 7 8 9 10 very relevant
High-quality Information on Mental Health Issues (comprehensible, professional)	not at all relevant 0 1 2 3 4 5 6 7 8 9 10 very relevant
Consulting Services / Online Communication with Experts (e.g. help line, e-mail support, discussion forum)	not at all relevant 0 1 2 3 4 5 6 7 8 9 10 very relevant
Communication Tools (e.g. closed discussion forum) for different stakeholder groups (e.g. teachers, pupils, students, clients, patients)	not at all relevant 0 1 2 3 4 5 6 7 8 9 10 very relevant

Suitability for Mobile Devices (smartphones, tablets)	not at all relevant 0 1 2 3 4 5 6 7 8 9 10 very relevant
Data Security	not at all relevant 0 1 2 3 4 5 6 7 8 9 10 very relevant

**11a.**	**Which <u>other characteristics</u> would you consider as very relevant?**
Open answer	

[E. Values and Attitudes]

**In your opinion, what are the three most important <u>advantages</u> and the three most important disadvantages of Internet-based prevention programs in the field of mental health, implemented in schools / universities / the health care system compared to face-to-face interventions?**

12. The three most important advantages:

1. *Open answer*
2. *Open answer*
3. *Open answer*

13. The three most important disadvantages:

1. *Open answer*
2. *Open answer*
3. *Open answer*

**14.**	**If you consider the advantages and disadvantages you have mentioned, how do you balance them in total?**
Slider	Much more disadvantages -5 -4 -3 -2 -1 0 1 2 3 4 5 Much more advantages

15.	Would you be in favor of integrating Internet-based prevention programs in the field of mental health in your school / your university / the health care system in your country?
Slider	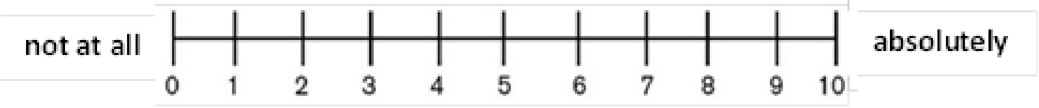

16.	Would you actively support the integration of such programs in your school / your university / the health care system in your country?
Slider	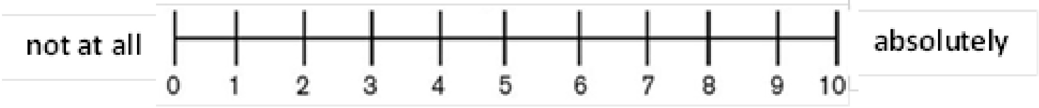

[F. Barriers and facilitators for reach, adoption, implementation, and maintenance]

The following questions focus on potential **hindering and fostering context factors** for different program phases (**1. Willingness for Implementation (Adoption), 2. Reach, 3. Implementation, 4. Maintenance)**. You are asked to provide your own ideas, suggestions and recommendations. Although some questions might be difficult for you to answer, please try to give a short answer to each of them, as your suggestions are very important for the successful implementation of the ICare project and other upcoming projects.

1. Willingness for implementation (ADOPTION)

By **Willingness for Implementation (Adoption)** we mean the proportion of schools and school personnel (e.g. teachers, school authorities, school psychologists, school physicians, school social workers, etc.) / universities and university personnel (e.g. university teachers, university authorities, university psychological services, university counselling services, university students association and groups, student representatives) / facilities and professionals in the health care system (e.g. general practitioners, psychiatrists, pediatricians, psychologists, psychotherapists, nurses) who are willing to implement a program.

What factors could influence the willingness for implementation (ADOPTION) of Internet-based prevention programs in the field of mental health in schools / universities / the health care system in your country?

17.	**Fostering factors for the willingness of implementation (adoption) in schools / universities / the health care system in your country:**
Open answer	

**18.**	**Hindering factors for the willingness of implementation (adoption) in schools / universities / the health care system in your country:**
Open answer	

2. REACH

By **Reach** we mean the proportion of pupils /university students / individuals of the target group(s) who are willing to participate in a programme.

What factors could influence the reach of Internet-based prevention programs in the field of mental health?

**19.**	**Fostering factors for the reach in schools / universities / the health care system:**
Open answer	

**20.**	**Hindering factors for the reach in schools / universities / the health care system:**
Open answer	

3. IMPLEMENTATION

By **Implementation** we mean that a programme is implemented as intended by different personnel in schools (e.g. teachers, school authorities, school psychologists, school physicians, school social worker, etc.) / universities (e.g. university teachers, university authorities, university psychological services, university counselling services, university students association and groups, student representatives, etc.) / the health care system (e.g. general practitioners, psychiatrists, pediatricians, psychologists, psychotherapists, nurses).

What factors could influence the implementation of Internet-based prevention programs in the field of mental health in schools / universities / the health care system in your country?

**21.**	**Fostering factors for the implementation in schools / universities / the health care system:**
Open answer	

**22.**	**Hindering factors for the implementation in schools / universities / the health care system:**
Open answer	

**23.**	**Who should be <u>responsible</u> for the IMPLEMENTATION of such programs in schools / universities / the health care system in your country?**
Open answer	

4. MAINTAINANCE

By **Maintenance** we mean the sustainability of the programme in schools / universities / the health care system (i.e. that the programme can be offered and used continuously).

What factors could influence the maintenance of Internet-based prevention programs in the field of mental health in schools / universities / the health care system in your country?

**24.**	**Fostering factors for the maintenance in schools / universities / the health care system:**
Open answer	

**25.**	**Hindering factors for the maintenance in schools / universities / the health care system:**
Open answer	

[G. Future research]

**26.**	**What would you suggest for further research regarding prevention programs in the field of mental health in general?**
Open answer	

Further Comments:

**27.**	**Is there anything else you would like to let us know?**
Open answer	

Thank you very much for your participation in this survey! Your responses have been saved. Please close the browser window.
